# A Web-Based Sexual Health Intervention to Prevent Sexually Transmitted Infections in Hong Kong: Model-Based Cost-Effectiveness Analysis

**DOI:** 10.2196/45054

**Published:** 2023-08-10

**Authors:** Wen Zhang, Carlos K H Wong, Yiqiao Xin, Daniel Y T Fong, Janet Y H Wong

**Affiliations:** 1 School of Nursing and Health Studies Hong Kong Metropolitan University Hong Kong China (Hong Kong); 2 Department of Pharmacology and Pharmacy Li Ka Shing Faculty of Medicine The University of Hong Kong Hong Kong China (Hong Kong); 3 Department of Family Medicine and Primary Care Li Ka Shing Faculty of Medicine The University of Hong Kong Hong Kong China (Hong Kong); 4 Health Economics and Health Technology Assessment University of Glasgow Glasgow United Kingdom; 5 School of Nursing The University of Hong Kong Hong Kong China (Hong Kong)

**Keywords:** economic evaluation, web-based intervention, young adult, cost-effectiveness, sexual health, sexually transmitted infection, STI, digital intervention

## Abstract

**Background:**

Sexually transmitted infections (STIs) remain a significant public health concern, particularly among young adults, and *Chlamydia trachomatis* (CT) infections are the most common STIs in young women. One of the most effective ways to prevent STIs is the consistent use of condoms during sexual intercourse. There has been no economic evaluation of the interactive web-based sexual health program, Smart Girlfriend, within the Chinese population.

**Objective:**

This study aimed to evaluate the long-term cost-effectiveness of Smart Girlfriend in preventing STIs in the Chinese population. The evaluation compared the program with a control intervention that used a 1-page information sheet on condom use.

**Methods:**

A decision-analytic model that included a decision tree followed by a Markov structure of CT infections was developed since CT is the most prevalent STI among young women. The model represents the long-term experience of individuals who received either the intervention or the control. One-way and probabilistic sensitivity analyses were conducted. The main outcomes were the number of CT infections and the incremental cost as per quality-adjusted life year (QALY).

**Results:**

A cohort of 10,000 sexually active nonpregnant young women initially entered the model in a noninfectious state (ie, “well”). In the base-case analysis, the implementation of the Smart Girlfriend program resulted in the prevention of 0.45% of CT infections, 0.3% of pelvic inflammatory disease, and 0.04% of chronic pelvic pain, leading to a gain of 70 discounted QALYs and cost savings over a 4-year time horizon, compared to the control group. With more than 4548 users, the intervention would be cost-effective, and with more than 8315 users, the intervention would be cost saving. A 99% probability of being cost-effective was detected with a willingness to pay US $17,409 per QALY.

**Conclusions:**

Smart Girlfriend is a cost-effective and possibly cost-saving program over a 4-year time horizon. This result was particularly sensitive to the number of website users; launching the website would be cost-effective if more than 4548 people used it. Further work is warranted to explore if the findings could be expanded to apply to women who have sex with women and in the context of other STIs.

**Trial Registration:**

ClinicalTrial.gov NCT03695679; https://clinicaltrials.gov/study/NCT03695679

## Introduction

Sexually transmitted infections (STIs), including *Chlamydia trachomatis* (CT), remain a significant public health concern both in Hong Kong and worldwide, particularly among young women, who are disproportionately affected [1,2]. According to data from the World Health Organization, it is estimated that over 1 million individuals worldwide are infected with STIs every day [3]. In the United States, young people aged 15-24 years account for half of all new infections reported. There were over 1.7 million reported cases of CT infections in 2017, which is the highest number ever reported for any type of infection (chlamydia: 528.8 cases per 100,000 population; gonorrhea: 171.9 cases per 100,000 population; and syphilis: 9.5 cases per 100,000 population) [4]. In Hong Kong, STIs are not locally notifiable, and we have only found 1 study reporting the population-based prevalence of CT in Hong Kong [5], which is 1.4%, higher than the prevalence of other common STIs in Hong Kong (eg, gonorrhea: 197.9 cases per 100,000 population and syphilis: 121.9 cases per 100,000 population) [6]. CT has also been reported to have the highest prevalence among young adult women (aged 18-26 years) in Hong Kong [5]. Although over 80% of women with genital chlamydia are asymptomatic, they can still be contagious, and the disease can increase the risk of long-term health consequences for women, including pelvic inflammatory disease (PID), ectopic pregnancy, infertility, and chronic pelvic pain (CPP) [7]. These consequences can have a notable influence on quality of life and lead to a high disease burden on the health care system. In the United States, the lifetime direct medical cost of CT had reached US $516.7 million in 2008 [4].

One of the most effective ways to prevent STIs is the consistent use of condoms during sexual intercourse [8], and laboratory studies have provided evidence that condoms provide a virtually impermeable barrier to prevent the transmission of CT [9]. However, previous evidence has indicated that only 17.2% of sexually active young Chinese women reported consistent condom use [10]. Furthermore, young Chinese women are especially vulnerable to inconsistent condom use due to their limited control over decisions regarding condom use [11]. Sexual health interventions in Hong Kong still lag far behind those in many other places. In Hong Kong, sexual health interventions always emphasize reproductive physiological knowledge or STI prevention, but they seldom focus on the effects of gender-related power dynamics, including sexual coercion, or on sexual health, respectful relationships, and sexual communication and negotiation [12]. An increasing body of evidence indicates that individuals who engage in more sexual communication and negotiation prior to sexual activity are more likely to report higher rates of condom use during intercourse [13]. Sexual coercion is known to be highly related to risky sexual behaviors, including inconsistent condom use. It has been recommended as a key concept in international technical guidance on sexuality education [14]. These facts underline the need to develop a comprehensive sexual health intervention in Hong Kong.

In 2018, we launched a sexual health program (Smart Girlfriend) for female university students in Hong Kong, which was an interactive web-based intervention. The website was designed to empower female college students to enhance their knowledge, attitudes, norms, and self-efficacy for managing their sexual health, including issues related to condom use, sexual coercion and sexual consent, casual sex, and sex communication. A multicenter randomized controlled trial (RCT) was carried out to evaluate the effectiveness of the Smart Girlfriend program among female college students with the primary outcome of enhancing condom use over a 6-month follow-up period, and results can be found elsewhere [15]. To the best of our knowledge, no studies have investigated the cost-effectiveness of a web-based sexual health intervention to prevent STIs in Hong Kong or other regions. A systematic review of digital interventions for sexual health promotion indicated that the cost-effectiveness of digital interventions is still unclear [16]. However, it is difficult to test the difference in quality-adjusted life years (QALYs) over a short-term follow-up period since the majority of patients with newly diagnosed STIs are asymptomatic [3] and the change in quality of life is not easily detectable over a short-term follow-up period. Therefore, in this study, we used a decision-analytic model to estimate the change in QALYs over a long-term follow-up period. We aimed to assess the cost-effectiveness of the Smart Girlfriend program in preventing STIs among young Chinese people over an extended time horizon. The decision-analytic model incorporates the transition between various disease stages over time. We hypothesized that the Smart Girlfriend program will be cost-effective if the incremental cost-effectiveness ratio (ICER) of QALYs is less than the cost-effectiveness threshold (CET) in Hong Kong. The CET in Hong Kong is estimated to be between HKD 133,915 and HKD 221,546 (US $17,409 to US $28,801) according to Wood et al [17].

## Methods

### Overview

A decision-analytic model was developed to test the cost-effectiveness of the Smart Girlfriend program (intervention) with a 1-page information sheet on condom use (control) in a cohort of sexually active Chinese women. A total of 781 sexually active Chinese emerging women who were unmarried, had been with intimate partners in the past 12 months, and had not received any sexual health information during the same period were recruited from 5 universities in Hong Kong. The participants were randomly allocated into either the intervention or the control group with a 1:1 randomization ratio through an embedded randomizer. The intervention group was authorized to access the entire content of the website that was developed based on the Continuum of Conflict and Control and Health Belief Model. The website aimed to prevent STIs by promoting consistent condom use among participants. The control group was given a 1-page information sheet through the website on how to use condoms correctly. All participants were authorized to access the corresponding information over 6 months. The details of the intervention can be found in a previous publication [15].

This cost-effectiveness analysis was conducted from a health care sector perspective, and a 3% annual discount rate was applied. The incremental cost per QALY was used to measure the comparative performance of the Smart Girlfriend program versus the control group. The model was designed and implemented in Microsoft Excel 2017. The CHEERS (Consolidated Health Economic Evaluation Reporting Standards) checklist was followed to report this study (Multimedia Appendix 1).

### Ethical Considerations

The behavioral data were obtained from the RCT (registered with ClinicalTrials.gov registry: NCT03695679), which had obtained ethics approval from the Institutional Review Board of the University of Hong Kong/Hospital Authority Hong Kong West Cluster (UW-17029). Written informed consent was received from the participants from the RCT. Participants who completed all of the web-based questions received coupons valued at HKD 300 (US $38.50). The study data are anonymous, and the participants’ privacy was protected. The information gathered from the survey was saved in a secure university database. Other data in this study were collected from the published literature and publicly accessed government websites without the need for ethical approval.

### Model

**Figure 1:**
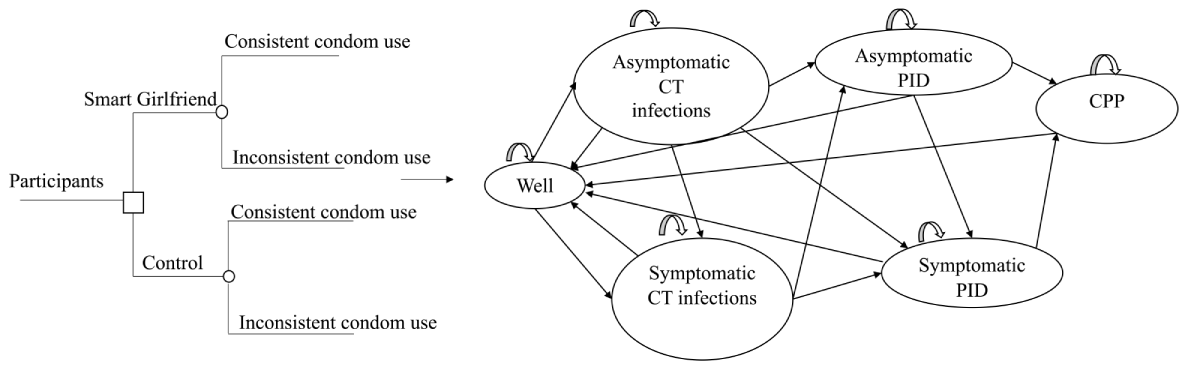
Model figure. CT: Chlamydia trachomatis; PID: pelvic inflammatory disease; CPP: chronic pelvic pain.

The model in this study focused on CT infection, as it is the most common STI in women [5]. The model consisted of a decision tree followed by a 6-state Markov structure (Figure 1). The health states included “well,” “asymptomatic CT infections,” “symptomatic CT infections,” “asymptomatic PID,” “symptomatic PID,” and “CPP.” The time horizon for the model was set at 4 years, with a 3-month cycle. A cohort of 10,000 sexually active nonpregnant young women entered the model, starting in a noninfectious state (ie, “well”). Uninfected women have the probability of developing a CT infection that might be asymptomatic or symptomatic. Asymptomatic infections could be spontaneously cured, become symptomatic over time, develop into PID, or remain asymptomatically infected. We assumed that women with symptoms would seek care and receive treatment. Similar to a CT infection, PID was modeled as symptomatic or asymptomatic, and asymptomatic PID was likely to become symptomatic. We estimated the possibility of asymptomatic PID or infections becoming symptomatic as 1% per cycle, with a sensitivity analyses range of 0% to 10% [18]. Patients with PID could develop long-term complications (eg, infertility, ectopic pregnancy, and CPP) and either remain in this state or be cured by effective treatment. In this model, we assumed that no women would become pregnant within the 4-year time horizon. Therefore, we only considered the proportion of PID cases that could potentially develop into CPP. Women who recover are usually subject to reinfection. We assumed there would be no deaths resulting from CT infections, PID, or their complications, as previous studies have reported very low overall mortality rates associated with CT infections, PID, and CPP [19]. In the base-case scenario, we assumed that the probabilities of disease progression to CT infections, PID, or CPP would remain constant over time and across different frequencies of condom use. The model focused exclusively on heterosexual intercourse. Furthermore, we assumed no secondary infection during the 4-year time horizon.

### Data

Behavioral data, including the frequency of sexual intercourse and condom use, were collected from the multicenter RCT at the 3-month postintervention follow-up (T1) and the 6-month postintervention follow-up (T2). The probability of consistent condom use (defined as 100% condom use) was calculated by using behavioral data (T1: intervention 69.4% and control 60.9%; T2: intervention 66.7% and control 62.7%). We assumed no intervention effect would be detected beyond 6 months in the intervention group, as previous research has indicated that web-based interventions do not result in long-term effects [20]; in other words, we assumed that the probability of consistent condom use in the intervention group would be the same as that in the control group (62.7%) after 2 cycles.

The probabilities, costs, and utilities used in this model and their plausible ranges are shown in Table 1 [18,21-34]. The transition probabilities were derived from published studies or specific data sources for China by searching PubMed, Google Scholar, and CNKI (China National Knowledge Infrastructure; a Chinese database). In cases where data on transition probabilities for Chinese patients with CT were not available, available data from other available regions were used. We estimated that 1% of asymptomatic CT infections would progress to symptomatic infections according to the previous model for CT [18], with sensitivity analyses varying from 0% to 10%. The probability of CT progressing to PID was determined based on a scoping review [21]. The proportion of CT infections being asymptomatic and the parameters of treatment efficacy of CT infections, PID, and CPP were derived from studies in the Chinese population [22-29]. Other parameters were derived from other countries, such as the United States [18,30], the Netherlands [31], and Austria [32], as no experimental study was reported for the Chinese population or Asian countries.

**Table 1:** Model parameters (baseline values and ranges used in sensitivity analyses). The number of sexually active nonpregnant young women who entered the model in the base-case scenario was 10,000. The discount rate was 0.03 and the plausible range was 0-0.05.

Category and parameter		Base case	Plausible range	Probabilistic sensitivity analyses			Reference, year
				Distribution	α	β	
**Transition probabilities and treatment efficacy**							
	Incidence of CT^a^ for consistent condom use (case per 100 person-years), n	8	6.4-9.6	Beta	22.2	1088	Rietmeijer et al [30], 2002
	Incidence of CT for inconsistent condom use (case per 100 person-years), n	15.2	12-18.4	Beta	82.6	2091	Rietmeijer et al [30], 2002
	Proportion of CT infections being asymptomatic, %	40.5	34.2-86	Beta	5.2	7.63	Chang et al [28], 2020; Detels et al [22], 2011; Yan et al [23], 2019
	Probability of asymptomatic CT infections or PID^b^ becoming symptomatic, %	1	0-10	Beta	.1	10	Smith et al [18], 2007
	Probability of spontaneously clearing CT infections, %	6.8	5-40	Beta	20	272	Smith et al [18], 2007; van Liere et al [31], 2019
	Probability of the treatment curing CT infections, %	82.6	74.2-94.2	Beta	76	16	Ai et al [25], 2016; Zhao [24], 2011
	Probability of CT infections progressing to PID, %	18	3.7-30	Beta	18	75	Haggerty et al [21], 2010
	Proportion of PID being symptomatic, %	40	15-48	Beta	14.6	21.9	Hu et al [33], 2004; Smith et al [18], 2007
	Probability of PID progressing to CPP^c^, %	9	7.5-11	Beta	205	2069	Walleser et al [32], 2006
	Probability of the treatment curing PID, %	82	66~90	Beta	33	7	Judlin et al [29], 2010
	Probability of the treatment curing CPP, %	72.7	56.5~79.1	Beta	39.4	14.8	Li et al [27], 2017; Qi et al [26], 2016
**Utility**							
	Well, asymptomatic CT infection, and asymptomatic PID	0.95	1-0.90	Beta	49.2	2.6	Lawrence et al [34], 2001
	CT infection (symptomatic)	0.75	0.65-0.85	Beta	55.5	18.5	Lawrence et al [34], 2001
	PID (symptomatic)	0.63	0.5-0.8	Beta	22.3	13.1	Lawrence et al [34], 2001
	CPP	0.6	0.5-0.8	Beta	21.9	14.6	Lawrence et al [34], 2001
**Cost per cycle (HKD)^d^**							
	CT infections	1190	950-1428	Gamma	1	1190	Multimedia Appendix 2
	PID	15,886	12,709-19,063	Gamma	1	15,886	Multimedia Appendix 2
	CPP	41,376	49,651-33,101	Gamma	1	41,376	Multimedia Appendix 2

^a^CT: *Chlamydia trachomatis*.

^b^PID: pelvic inflammatory disease.

^c^CPP: chronic pelvic pain.

^d^Currency: HKD 1=US $0.13, as of April 16, 2021.

The cost of developing and maintaining the website (HKD 120,000) was collected from the Smart Girlfriend program. The cost of the 1-page leaflet (HKD 0.2/piece) was collected from a local print service provider in Hong Kong. The direct cost of diseases in Hong Kong was estimated according to the Centre for Health Protection Guidelines of the Government of Hong Kong, MIMS Hong Kong, and previous literature [35-39]. Details on the estimation of the direct medical cost of diseases can be found in Multimedia Appendix 2. The unit prices were derived from the Hospital Authority in Hong Kong [40]. The estimated costs were subjected to ±20% variation in the sensitivity analyses. It was assumed that CPP would occur 6 months after PID, as CPP is defined as persistent, noncyclic pain lasting over 6 months and associated with pelvis structures [41]. Future costs were discounted at an annual rate of 3%.

### Cost-Effectiveness Analysis

The result of the cost-effectiveness analysis was presented as ICER, which is defined as the incremental cost of achieving 1 unit of a health outcome (ie, QALYs gained). The ICER was calculated as the difference in health outcomes divided by the difference in total costs, as follows:



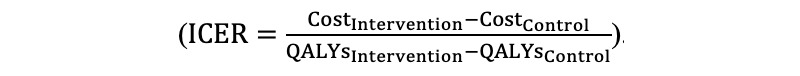



CET is commonly used to determine if an intervention is worthwhile and should represent the cost of health opportunity. In this study, the intervention was considered cost-effective compared to the control group if the ICER was lower than 1 to 3 times Hong Kong’s gross domestic product per capita, as suggested by the World Health Organization [42]. Additionally, the CET was set at HKD 133,915 to HKD 221,546 (US $17,409 to US $28,801), accounting for opportunity costs [17]. The Smart Girlfriend program was considered cost-effective when the ICER was less than the CET. If the future costs are saved as a result of QALYs gained, the Smart Girlfriend program will be considered a cost-saving program [43].

### Sensitivity Analyses

One-way sensitivity analyses were conducted to explore the robustness of the base-case results by changing the parameters one at a time (Table 1). Threshold values of the parameters were calculated to determine the point at which the Smart Girlfriend program becomes a cost-saving or cost-effective program compared to the control group. In addition, a probabilistic sensitivity analysis was performed using a Monte Carlo simulation with 5000 random iterations from the distribution assigned to the model parameters, which allowed all variables to vary simultaneously. A beta distribution was chosen for transition probabilities and utilities, and a gamma distribution was chosen for costs (Table 1). The uncertainty of the ICER estimate was illustrated using a cost-effectiveness plane. The cost-effectiveness acceptability curve was derived from the probabilistic sensitivity analysis and represented the probability that the intervention (or the control) would be considered cost-effective across different willingness-to-pay thresholds. The incremental net monetary benefit statistic was also estimated, and a positive value indicated that the intervention was cost-effective. In this study, the exchange rate was HKD 1=US $0.13, as of April 16, 2021.

## Results

### Base-Case Analyses

**Figure 2:**
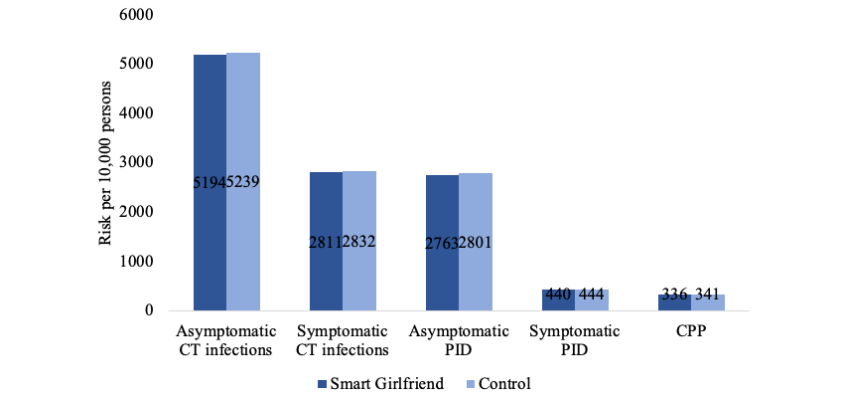
Estimated risks in the Smart Girlfriend program and the control group. CPP: chronic pelvic pain; CT: *Chlamydia trachomatis*; PID: pelvic inflammatory disease.

The estimated risks of getting asymptomatic CT infections, symptomatic CT infections, asymptomatic PID, symptomatic PID, and CPP over a 4-year time horizon are presented in Figure 2. Compared to the control group, the intervention was estimated to avert 0.45% of CT asymptomatic infections (intervention: 7934 per 10,000 persons vs control: 7979 per 10,000 persons), 0.3% of PID cases (intervention: 3178 per 10,000 persons vs control: 3208 per 10,000 persons), and 0.04% of CCP cases (intervention: 333 per 10,000 persons vs control: 337 per 10,000 persons).

The baseline cost-effectiveness results showed that the Smart Girlfriend program was cost saving in comparison to the control group (Table 2). The base-case analyses showed that the intervention generated cost savings of HKD 33,400 (US $4342) per 10,000 participants enrolled in the program. Therefore, the Smart Girlfriend program dominated in terms of both cost and effectiveness.

**Table 2:** Cost-effectiveness results for the base-case analysis. Costs and outcomes, that is, quality-adjusted life years (QALYs), are reported per person.

Strategy	Cost (HKD^a^)	Incremental costs (HKD)	QALYs	Incremental QALYs	ICER^b^ (HKD)
Smart Girlfriend	2237.99	N/A^c^	3.5517	N/A	Cost-saving
Control group	2241.33	–3.34	3.5516	0.0001	N/A

^a^Currency: HKD 1=US $0.13, as of April 16, 2021.

^b^ICER: incremental cost-effectiveness ratio.

^c^N/A: not applicable.

### One-Way Sensitivity Analyses

**Figure 3:**
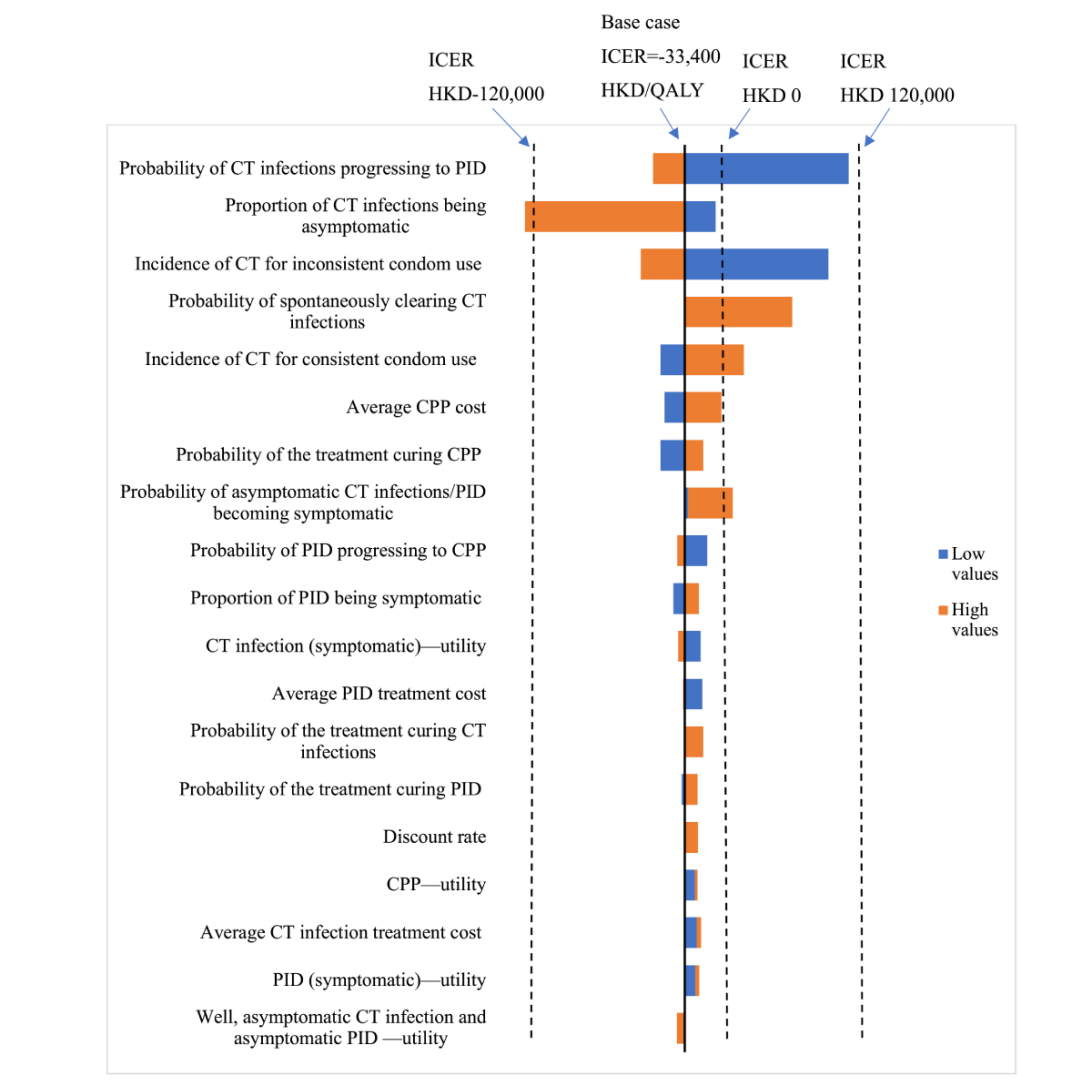
One-way sensitivity analysis. Currency: HKD 1=US $0.13. CT: *Chlamydia trachomatis*; PID: pelvic inflammatory disease; CPP: chronic pelvic pain.

In one-way sensitivity analyses, the results (Figure 3) showed that 5 key parameters had a large effect on the ICER and impacted the dominance of the Smart Girlfriend program over the control group when the Smart Girlfriend was no longer cost saving. The 5 key parameters included (1) the incidence of CT infections for consistent condom use, (2) the incidence of CT infections for inconsistent condom use, (3) the probability of asymptomatic CT infections or PID becoming symptomatic, (4) the probability of spontaneously clearing CT infections, and (5) the probability of CT infections progressing to PIDs. Other parameters had a limited effect on the results. When a CET of HKD 133,915 (US $17,409) was applied as the willingness-to-pay threshold for a 1-unit increase in QALYs, the Smart Girlfriend program was deemed cost-effective across a plausible range of values for all these parameters.

**Figure 4:**
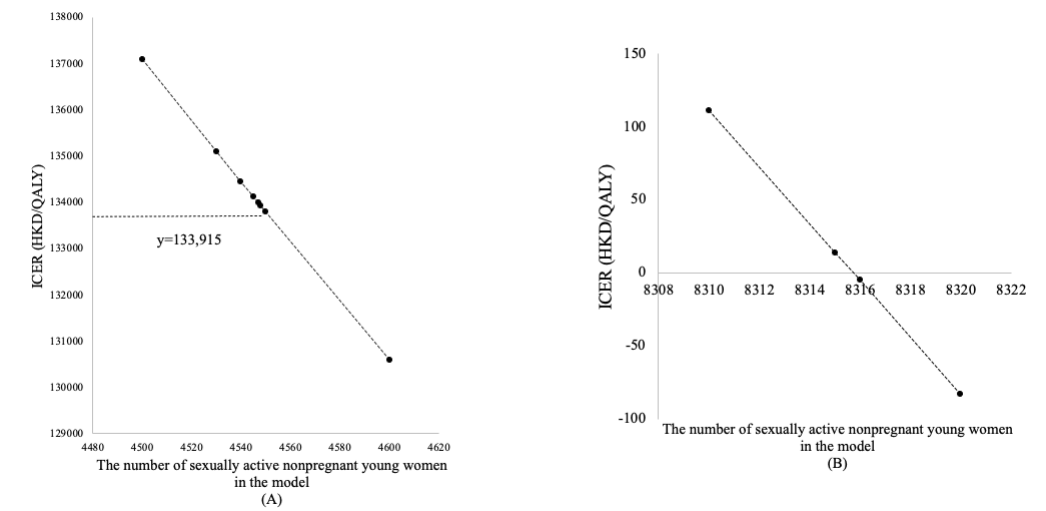
One-way sensitivity analysis for the number of sexually active nonpregnant young women who entered the model. Currency: HKD 1=US $0.13. ICER: incremental cost-effectiveness ratio; QALY: quality-adjusted life year.

Additionally, a threshold analysis showed the impact of varying the number of sexually active nonpregnant young women entering the model on the ICER, as it would affect the per-person intervention cost related to website development and maintenance. When the cohort size exceeded 4548, the Smart Girlfriend program was cost-effective over a 4-year time horizon at a willingness-to-pay threshold of HKD 133,915 (US $17,409; Figure 4A). When the cohort size exceeded 8315, the Smart Girlfriend program was cost saving (Figure 4B).

### Probabilistic Sensitivity Analysis

**Figure 5:**
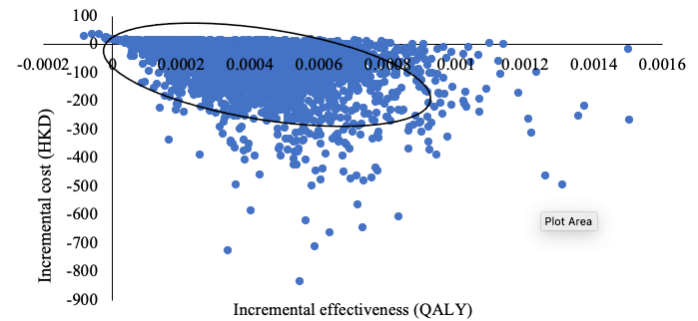
Cost-effectiveness plane with Monte Carlo simulations (5000 iteration). Currency: HKD 1=US $0.13. QALY: quality-adjusted life year.

**Figure 6:**
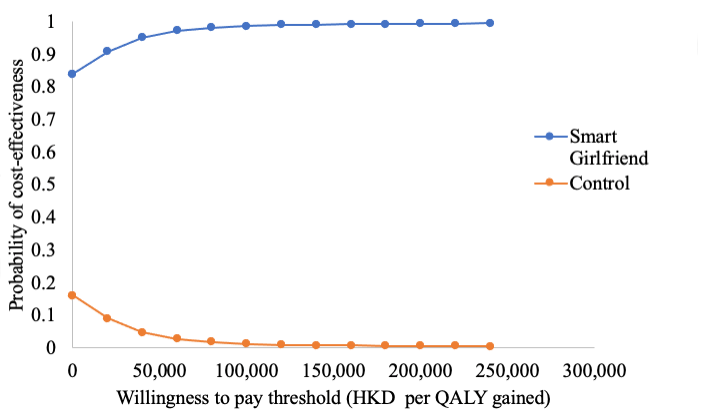
Cost-effectiveness acceptability curves. Currency: HKD 1=US $0.13. QALY: quality-adjusted life year.

The results of probabilistic sensitivity analysis are shown in Figures 5 and 6. In the cost-effectiveness plane (Figure 5), each dot represents a pair of incremental costs and incremental QALYs resulting from the 5000 Monte Carlo simulations. The expected incremental cost was HKD –57.26 (SD 76.82) and the expected incremental QALYs was 0.0004 (SD 0.0002). The incremental net monetary benefit was 108.94 (95% CI 111.44-106.44).

A total of 83.8% (4190/5000) of the ICERs were located in the fourth quadrant of the coordinate axis on the graph, implying that the Smart Girlfriend program was cost saving, resulting in lower costs and more QALYs compared to the control group. Only 8 (0.2%) simulations fell within the left quadrants of the graph, implying higher QALYs with the control strategy. The cost-effectiveness acceptability curve showed that the Smart Girlfriend program had a 99% probability of cost-effectiveness at a willingness-to-pay threshold of HKD 133,915 (US $17,409) per QALY (Figure 6).

## Discussion

### Principal Findings

To our best knowledge, this is the first study to explore the cost-effectiveness of a web-based sexual health intervention to prevent STIs. This study found that the Smart Girlfriend program was cost saving in preventing CT compared to a 1-page information sheet about condom use over a 4-year time horizon, although the difference in costs and QALYs was very small. The sensitivity analyses showed that the Smart Girlfriend program had a 99% probability of cost-effectiveness at a willingness-to-pay threshold of HKD 133,915 (US $17,409) per QALY. This finding was robust across a wide range of plausible parameter input values specific to the Hong Kong Chinese population, including higher costs and a lower CT incidence. This conclusion is in line with a study by Burgos et al [44] who found that their 35-minute behavioral intervention for female sex workers would be cost-effective in reducing STIs over a long-term time horizon, with US $183 per additional QALY gained. Our findings suggest that the Smart Girlfriend program is an attractive option for decision makers from a cost-effectiveness perspective, yielding a stream of net savings starting approximately 4 years after implementation.

The per-person cost of the intervention was highly dependent on the number of individuals using the website, as the total costs of developing and maintaining a website would remain the same regardless of the number of people visiting the website. We found that if more than 4548 users used the website, Smart Girlfriend would be considered cost-effective, and if more than 8315 users used the website, it would be cost saving. Additionally, we have demonstrated in our previous study [15] that the Smart Girlfriend website was widely accepted among young women. These figures strongly suggest that it would be feasible for the Smart Girlfriend program to achieve cost-effectiveness, and policy makers should consider promoting this type of program in Hong Kong.

### Strengths and Limitations

This study has several strengths. First, this model-based economic evaluation was conducted in the Chinese context. China-specific demographic, epidemiological, and direct medical cost data were used whenever possible. We estimated the direct medical costs of CT, PID, and CPP treatments in Hong Kong. Some previous models for preventing CT have been applied in other counties, such as the United States [18] and the Netherlands [45]. The model parameters in these studies were derived from non-Chinese populations, which might not accurately represent the Chinese population; additionally, these studies did not evaluate the cost-effectiveness of a brief digital support intervention for promoting sexual health. This study may contribute valuable evidence on the cost-effectiveness of a brief digital support intervention for promoting sexual health in the Chinese population. The potential values of this digital support extend to Chinese populations elsewhere, as comprehensive sensitivity analyses resulted in robust conclusions across a wide range of plausible parameter input values. However, caution should be exercised when extending the conclusions to other cultural adaptations of this digital support prior to any further evidence. Second, this study estimated cost-effectiveness over a long-term time horizon using a Markov model. In previous trial-based cost-effectiveness research on sexual health interventions [46], the loss of QALYs might have been underestimated because there could be a potential for loss of QALYs as CT infections progressed to PID.

Several limitations in this study should be considered when interpreting the findings. We assumed that all sexual relations were heterosexual, as there were no data available for the risks of getting CT infections among women who have sex with women. In the future, when more empirical data on homosexual women are available, this model could be expanded to homosexual women. We made some assumptions to make this model feasible and simple. We did not consider the secondary infections, when infection occurs during or after treatment for another infectious disease, nor did we consider the risks of participants’ partners developing a CT infection. Furthermore, we only focused on one of the most common STIs among women, although consistent condom use is also beneficial for preventing other STIs, such as HIV. In general, the estimated cost-effectiveness would be much higher if the potential benefits of preventing other STIs, the averted infections of sexual partners, and the secondary infections were included. Hence, it should be noted that the current model, based on the assumptions made, yields conservative estimates of infections averted. Additionally, we assumed that women with symptoms would seek care and treatment. Although a high care-seeking behavior was reported in patients with PID and CCP, this assumption may introduce bias in our results.

### Conclusions

In summary, this study shows that the Smart Girlfriend program is cost-effective and possibly cost saving. The cost-effectiveness of the program was particularly sensitive to the number of people who used the website. Launching the website would be cost-effective if more than 4548 individuals used it. Further work is needed to elucidate the cost-effectiveness of the program among women who have sex with women.

## Appendix

Multimedia Appendix 1 The CHEERS (Consolidated Health Economic Evaluation Reporting Standards) checklist used in the study.

Multimedia Appendix 2 Estimate of costs.

## Abbreviations

CET: cost-effectiveness threshold
CHEERS: Consolidated Health Economic Evaluation Reporting Standards
CNKI: China National Knowledge Infrastructure
CPP: chronic pelvic pain
CT: *Chlamydia trachomatis*
ICER: incremental cost-effectiveness ratio
PID: pelvic inflammatory disease
QALY: quality-adjusted life year
RCT: randomized controlled trial
STI: sexually transmitted infection

## Data Availability

The behavioral data sets analyzed during this study are not publicly available because of ethical requirements but are accessible upon reasonable request from the corresponding author. Other data analyzed during this study can be found in this paper and in the multimedia appendices.
