# Supplementary material for: A Web-Based Sexual Health Intervention to Prevent Sexually Transmitted Infections in Hong Kong: Model-Based Cost-Effectiveness Analysis
Source: J Med Internet Res. 2023 Aug 10;25:e45054. doi: 10.2196/45054 (PMC10450529; doi:10.2196/45054)
Supplement: Multimedia Appendix 1 [file jmir_v25i1e45054_app1.pdf]

The CHEERS 2022 checklist.

Section/topic	Item No	Guidance for reporting	Reported in section
**Title**			
Title	1	Identify the study as an economic evaluation and specify the interventions being compared.	Title Page, Paragraph 1
**Abstract**			
Abstract	2	Provide a structured summary that highlights context, key methods, results, and alternative analyses.	Abstract, Paragraphs 1–4
**Introduction**			
Background and objectives	3	Give the context for the study, the study question, and its practical relevance for decision making in policy or practice.	Introduction, Paragraphs 1–3
**Methods**			
Health economic analysis plan	4	Indicate whether a health economic analysis plan was developed and where available.	Methods, Overview Paragraph 1, and Model Paragraph 1

Section/topic	Item No	Guidance for reporting	Reported in section
Study population	5	Describe characteristics of the study population (such as age range, demographics, socioeconomic, or clinical characteristics).	Methods, Model, Paragraph 1
Setting and location	6	Provide relevant contextual information that may influence findings.	Methods, Overview, Paragraph 1
Comparators	7	Describe the interventions or strategies being compared and why chosen.	Methods, Overview, Paragraph 1 and Introduction, Paragraph 3
Perspective	8	State the perspective(s) adopted by the study and why chosen.	Methods, Overview, Paragraph 1
Time horizon	9	State the time horizon for the study and why appropriate.	Methods, Model, Paragraph 1
Discount rate	10	Report the discount rate(s) and reason chosen.	Methods, Overview, Paragraph 1
Selection of outcomes	11	Describe what outcomes were used as the measure(s) of benefit(s) and harm(s).	Methods, Data, Paragraph 1, and Appendix 2
Measurement of outcomes	12	Describe how outcomes used to capture benefit(s) and harm(s) were measured.	Methods, Data, Paragraph 1, Appendix 2, Cost-effectiveness analysis, Paragraph 1.

Section/topic	Item No	Guidance for reporting	Reported in section
Valuation of outcomes	13	Describe the population and methods used to measure and value outcomes.	Methods, Data, Paragraph 1-3, and Appendix 2
Measurement and valuation of resources and costs	14	Describe how costs were valued.	Methods, Data, Paragraph 1-3, and Appendix 2
Currency, price date, and conversion	15	Report the dates of the estimated resource quantities and unit costs, plus the currency and year of conversion.	Methods, Sensitivity analyses, Paragraph 1
Rationale and description of model	16	If modelling is used, describe in detail and why used. Report if the model is publicly available and where it can be accessed.	Methods, Model, Paragraph 1, Figure 1
Analytics and assumptions	17	Describe any methods for analysing or statistically transforming data, any extrapolation methods, and approaches for validating any model used.	Methods, Model, Paragraphs 1
Characterising heterogeneity	18	Describe any methods used for estimating how the results of the study vary for subgroups.	Not applicable

Section/topic	Item No	Guidance for reporting	Reported in section
Characterising distributional effects	19	Describe how impacts are distributed across different individuals or adjustments made to reflect priority populations.	Not applicable
Characterising uncertainty	20	Describe methods to characterise any sources of uncertainty in the analysis.	Methods, Statistical analysis, Paragraph 1
Approach to engagement with patients and others affected by the study	21	Describe any approaches to engage patients or service recipients, the general public, communities, or stakeholders (such as clinicians or payers) in the design of the study.	Not applicable
**Results**			
Study parameters	22	Report all analytic inputs (such as values, ranges, references) including uncertainty or distributional assumptions.	Methods, Table 1<br>Appendix 2
Summary of main results	23	Report the mean values for the main categories of costs and outcomes of interest and summarise them in the most appropriate overall measure.	Results, Base-case analyses, Paragraphs 1–2, Table 2 and Figure 2

Section/topic	Item No	Guidance for reporting	Reported in section
Effect of uncertainty	24	Describe how uncertainty about analytic judgments, inputs, or projections affect findings. Report the effect of choice of discount rate and time horizon, if applicable.	Results, One-way sensitivity analyses, Paragraphs 1-2, Figure 3 and Figure 4; Probabilistic sensitivity analysis, Paragraphs 1-2, Figure 5 and Figure 6.
Effect of engagement with patients and others affected by the study	25	Report on any difference patient/service recipient, general public, community, or stakeholder involvement made to the approach or findings of the study	Not applicable
**Discussion**			
Study findings, limitations, generalisability, and current knowledge	26	Report key findings, limitations, ethical or equity considerations not captured, and how these could affect patients, policy, or practice.	Discussion, Paragraphs 1–5
**Other relevant information**			
Source of funding	27	Describe how the study was funded and any role of the funder in the identification, design, conduct, and reporting of the analysis	Funding/Support, Paragraph 1

Section/topic	Item No	Guidance for reporting	Reported in section
Conflicts of interest	28	Report authors conflicts of interest according to journal or International Committee of Medical Journal Editors requirements.	Supplementary material
