# Supplementary material for: A Web-Based Sexual Health Intervention to Prevent Sexually Transmitted Infections in Hong Kong: Model-Based Cost-Effectiveness Analysis
Source: J Med Internet Res. 2023 Aug 10;25:e45054. doi: 10.2196/45054 (PMC10450529; doi:10.2196/45054)
Supplement: Multimedia Appendix 2 [file jmir_v25i1e45054_app2.docx]

**Appendix Estimates of costs**

Table 1 Unite cost

| Items | Cost (HKD) | Cost(USD)^e^ |
| --- | --- | --- |
| **Healthcare Services** |  |  |
| Clinic visit ^a^ |  |  |
| General Clinic Visit | 445/ visit | 58/ visit |
| Specialist Clinic Visit (Gynecologist) | 1190/ visit | 155/ visit |
| General ward ^a^ | 5100/ day | 663/ day |
| Injection ^a^ | 100/ attendance | 13/ attendance |
| Pelvic ultrasonography ^b^ | 3,605 | 469 |
| Diagnostic laparoscopy ^b^ | 11,570^*^ | 1504 |
| Molecular detection of Chlamydia trachomatis ^c^ | 220/ test | 29 |
| **Drug cost ^d^** |  |  |
| Ceftriaxone | 80.8(/250mg IM) |  |
| Ceftriaxone | 4.85(/1g IV) |  |
| Doxycycline | 1.33(100mg tab.) |  |
| Metronidazole | 0.12(400mg tab.) |  |

Note: a. Data were from 2017 Hong Kong SAR Government Gazette and Hospital Authority Ordinance (chapter 113): Public charges – non-eligible persons.

b. Data were from 2017 Hong Kong SAR Government Gazette and Hospital Authority Ordinance (chapter 113): Private charges (mid-point value).

c. This price is from Fees and charges for Public Health Care Service provided by the Department of Health, Hong Kong SAR Government.

d. Data were from the Document from Centre for Health Protection: Reducing bacterial resistance with IMPACT [40].

e. In this study, the currency was 1 HKD = 0.13 US, on 16 April 2021.

Table 2 Estimated direct medical costs

| Items | Cost (HKD) | Cost (USD) |
| --- | --- | --- |
| Average direct medical cost of CT infections ^a^ | 1,190 | 155 |
| Average direct medical cost of PID ^b^ | 15,886 | 2,065 |
| Average direct medical cost of CPP ^c^ | 41,376 | 5,379 |

Note: a. The treatment cost is a package price released in the government website of Hong Kong for non-eligible patients. (<https://www.dh.gov.hk/english/useful/useful_fee/useful_fee_os.html>)

b. According to the previous literature [36], it was assumed that 75% of symptomatic PID is treated on an outpatient basis which involves 2 GP visits, a test for C trachomatis. The inpatient PID was remaining 25% and was involved 9.6 days for admission, 1 GP visit and diagnostic laparoscopy and medication. According to the guidelines of PID [37, 38], the treatment for PID outpatients was ceftriaxone 250mg (IM) and Doxycycline 100mg orally for 14 days with Metronidazole 400mg orally 3 times daily for 14 days; the treatment for PID inpatient was Ceftriaxone 2g (IV) every 24 hours and Doxycycline 100mg orally and Metronidazole 400mg orally twice a day to complete 14 days.

c. Based on the previous literature [36], the treatment of CPP was assumed to include pelvic ultrasonography, and a 75% possibility of laparoscopy. Because of no reliable information, no surgery was considered. Since the analgesics are taken when patients request, analgesics should not be used for a prolonged period [35, 39]. The costs of analgesics were not included based on a conservative assumption. It was assumed that 70% of CPP is treated on an outpatient basis and 30% of CPP is treated on an inpatient basis [36]. The treatment for CPP outpatient was assumed to include 10 appointments with a physician, including 3 GP visits and 7 gynecologists. The inpatient care was assumed to involve 2 appointments with a gynecologist, 9.6 days for admission proceed by 1 GP visit and pelvic ultrasonography and laparoscopy.

IM refers to Intramuscular Injection; IV refers to Intravenous Injection; GP refers to General Practitioner; CT refers to Chlamydia Trachomatis; PID refers Pelvic Inflammatory Disease; CPP refers to Chronic Pelvic Pain.

**Reference in Appendix VI**

35. MIMIS Online. URL: https://specialty.mims.com/chronic%20pelvic%20pain%20in%20women/

treatment?channel=obstetrics-gynaecology [accessed 2020-08-31]

36. Postma MJ, Welte R, van den Hoek JAR, van Doornum GJ, Jager H, Coutinho RA. Cost-effectiveness of partner pharmacotherapy in screening women for asymptomatic infection with Chlamydia Trachomatis. Value Health 2001;4(3):266-275 [doi: 10.1046/j.1524-4733.2001.43009.x] [Medline: 11705188]

37. Ngu S, Cheung V. An update on the management of acute pelvic inflammatory disease. Hong Kong Medical Diary 2011;16(10):9-11

38. Ross J, Guaschino S, Cusini M, Jensen J. 2017 European guideline for the management of pelvic inflammatory disease. Int J STD AIDS 2018 Feb;29(2):108-114 [doi: 10.1177/0956462417744099] [Medline: 29198181]

39. Vincent K, Evans E. An update on the management of chronic pelvic pain in women. Anaesthesia 2021 Apr;76 Suppl 4:96-107 [doi: 10.1111/anae.15421] [Medline: 33682093]

40. Ho P, Wu T, Chao D. Reducing bacterial resistance with IMPACT. 2017. URL: http://www.chp.gov.hk/files/pdf/reducing_bacterial_resistance_with_impact.pdf [accessed 2023-07-17]
